# Lithium-ion conducting oxide single crystal as solid electrolyte for advanced lithium battery application

**DOI:** 10.1038/s41598-018-27851-x

**Published:** 2018-07-02

**Authors:** Kunimitsu Kataoka, Hiroshi Nagata, Junji Akimoto

**Affiliations:** National Institute of Advanced Industrial Science and Technology (AIST), 1-1-1 Higashi, Tsukuba, Ibaraki 305-8565 Japan

## Abstract

Today, all-solid-state secondary lithium-ion batteries have attracted attention in research and development all over the world as a next-generation energy storage device. A key material for the all-solid-state lithium batteries is inorganic solid electrolyte, including oxide and sulfide materials. Among the oxide electrolytes, garnet-type oxide exhibits the highest lithium-ion conductivity and a wide electrochemical potential window. However, they have major problems for practical realization. One of the major problems is an internal short-circuit in charging and discharging. In the polycrystalline garnet-type oxide electrolyte, dendrites of lithium metal easily grow through the void or impurity in grain boundaries of the sintered body, which causes serious internal short-circuits in the battery system. To solve these problems, we present an all-solid-state battery system using a single-crystal oxide electrolyte. We are the first to successfully grow centimeter-sized single crystals of garnet-type by the floating zone method. The single-crystal solid electrolyte exhibits an extremely high lithium-ion conductivity of 10^−3^ S cm^−1^ at 298 K. The garnet-type single-crystal electrolyte has an advantageous bulk nature to realize the bulk conductivity without grain boundaries such as in a sintered polycrystalline body, and will be a game-changing technology for achieving highly safe advanced battery systems.

## Introduction

Recently, research and development on all-solid-state secondary lithium-ion batteries (LIBs) are being actively carried out as next-generation batteries in order to realize high energy density, high power density, and high safety^1–3^. The all-solid-state LIBs using non-flammable inorganic solid electrolytes including oxides and sulfides are superior in safety to the current LIBs using flammable organic liquid electrolytes. In addition, the inorganic solid electrolytes may be able to utilize high potential positive electrode materials that could not be used due to the decomposition of the organic electrolyte. There is also the possibility of using metallic lithium that had a short-circuit problem. Furthermore, inorganic solid electrolytes are not flammable like organic liquid electrolytes. Although these perceptions are widely supported, inorganic solid electrolytes have not yet been put into practical use because the lithium-ion conductivity is lower than that of organic liquid electrolytes. For this reason, practical inorganic solid electrolytes are currently being actively developed in the development of all-solid-state LIBs. In inorganic solid electrolyte materials, glass and crystalline sulfides have high lithium-ion conductivity^4,5^, but they have the risk of hydrogen sulfide being generated upon air exposure. Also, metallic lithium could not be used as an anode because of reactivity with sulfide solid electrolytes. In contrast, oxide solid electrolytes have excellent safety, but the lithium-ion conductivity (10^−4^ S cm^−1^) is one order of magnitude lower than sulfide conductivity^6–16^. We began developing all-solid-state LIBs using oxide solid electrolytes for safety. In oxide electrolyte materials, perovskite-type^6^, NASICON,-type^7^ and garnet-type^8–16^ are well known framework host structures. We focused on garnet-type oxide that demonstrates the highest total lithium-ion conductivity among the oxide electrolytes. In addition, the garnet-type oxide electrolyte has a wide electrochemical potential window and is really suitable for achieving high energy density battery systems using a combination of the 5 V-class cathode and metallic lithium anode. Among the garnet-type oxide compounds, Li_7−x_La_3_Zr_2−x_Nb_x_O_12_ (LLZNb)^8,9^ and Li_7−x_La_3_Zr_2−x_Ta_x_O_12_ (LLZTa)^10–12^, in which a part of the zirconium site in Li_7_La_3_Zr_2_O_12_ (LLZ)^13–15^ is substituted with niobium and/or tantalum, are very attractive because they have the highest lithium-ion conductivity and a wide potential window. Recently, there are many reports that LLZ of the garnet-type solid electrolyte partially replaced with Ga and/or Sc and Al has high ionic conductivity of 10^−3^ order^17–19^. Furthermore, it has been reported that the space group changes from the ordinary *Ia*-3*d* to *I*-43*d* in the system in which Ga is substituted from the result of detailed single crystal structure analysis^20^. However, the garnet-type oxide electrolytes have major problems to being improved. One is a further improvement of the above lithium ion conductivity at room temperature. The other is internal short-circuits when charging^3^. In the polycrystalline garnet-type oxide electrolyte, lithium metal dendrites easily grow through the void or impurity in grain boundaries of the sintered body, which causes serious internal short-circuits in the battery system^9,10,12,21^. In order to solve this problem, it is necessary to improve the sintered density of the polycrystalline body. Ultimately, it is desirable to use a single-crystal body because the relative density of single crystals is 100% (i.e., there is no void). In addition, since there is no void or grain boundary in a logical single-crystal body, it is possible to produce an electrolyte with only bulk conductivity. However, only very small single crystals (less than 1 mm size of LLZ) have been prepared in the literature by a flux method or high-temperature heating, and electrochemical properties of the bulk garnet-type oxide compounds have not been determined yet^14–16^. In general, several inch-sized large single crystals of inorganic functional materials can be manufactured by using a melt growth technique such as the Czochralski (CZ) method and the floating zone (FZ) method. However, there are no reports on the high temperature phase diagram for LLZNb. Moreover, additional care should be taken to prevent decomposition of the garnet structure due to volatilization of lithium from the melt. Therefore, we first attempted to grow large single-crystals of LLZNb by zone melting technique using an FZ apparatus. In addition, we investigated the detailed chemical, structural, and electrochemical properties of LLZNb using the single-crystal samples. Finally, we demonstrated the performance of the all-solid-state lithium batteries using the single-crystal electrolyte.

In order to obtain centimeter-sized single crystal rods of Li_7−x_La_3_Zr_2−x_Nb_x_O_12_ (x = 0.2, 0.35, 0.45, 0.5, 0.6, 0.8), we optimized growth conditions by the FZ method. In the subsequent crystal growth experiments, we used feed rods with 20% excess lithium content. Even in these cases, the melting zone could not be stabilized, probably because too much lithium volatilized at high temperatures. Therefore, we moved the feed rod at higher speed with higher rotation speed in air flow to stabilize the melting zone.

**Figure 1:**
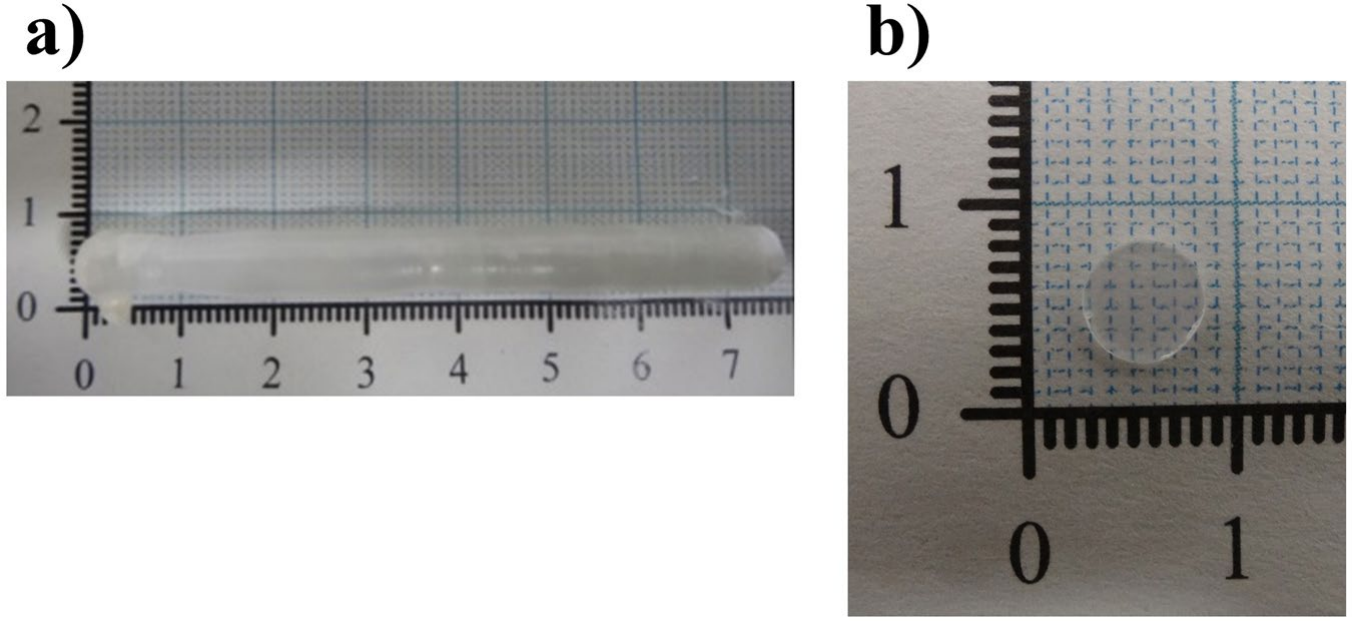
(**a**) As-grown LLZNb05 single-crystal rod and (**b**) A polished LLZNb05 single-crystal plate.

**Figure 2:**
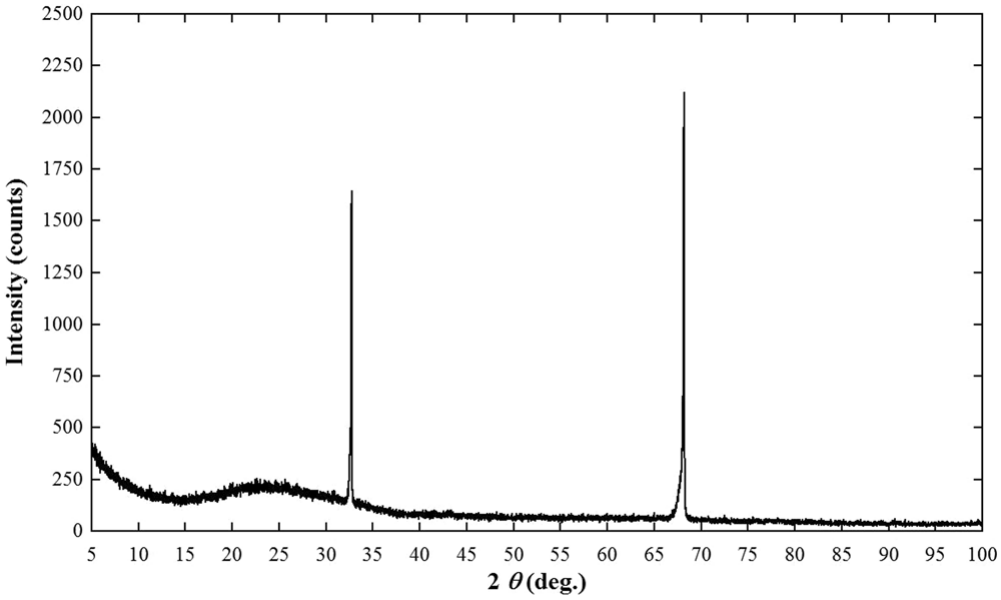
X-ray diffraction of the single-crystal plate by two axis X-ray diffractometer.

**Figure 3:**
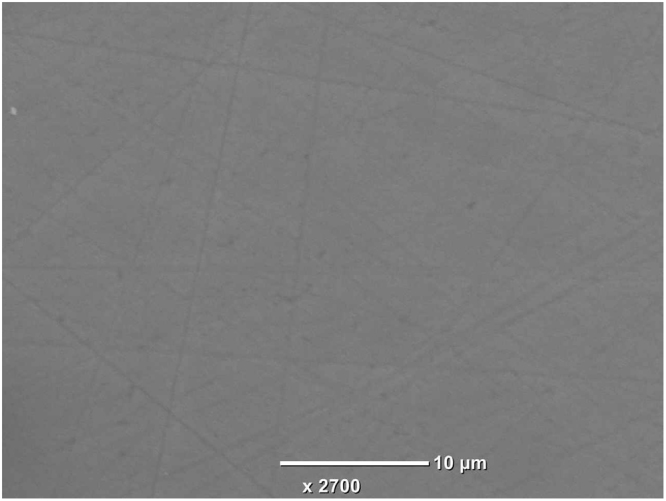
Photograph of the surface of a single-crystal plate by scanning electron microscope.

Figure 1(a) shows a typical single-crystal rod of Li_6.5_La_3_Zr_1.5_Nb_0.5_O_12_ (LLZNb05) about 8 mm in diameter and 60 mm length. Figure 1(b) shows a typical single-crystal plate polished on the surface and the outer periphery after slicing from the single-crystal rod. The size is about 6 mm in diameter and 0.7 mm in thickness. The bulk part of the crystal was colorless and transparent, while the outside of the crystal was cloudy but transparent. Figure 2 presents X-ray diffraction of the single-crystal plate by two axis X-ray diffractometer. This figure indicated the crystal growth direction was <332>. It is clear from the structure refinement in the latter section that the {332} lattice plane corresponds to a plane that traverses the diffusion path of lithium ions in the garnet-type framework structure. Figure 3 shows a image on the surface of a single crystal plate by scanning electron microscope (SEM). Only the polishing scratches were observed on the crystal surface; voids and grain boundaries were not observed. Chemical analysis by ICP-AES using approximately 50 mg of the crystals determined the atomic ratio of Li:La:Zr:Nb to be 6.5:3:1.5:0.5.

**Figure 4:**
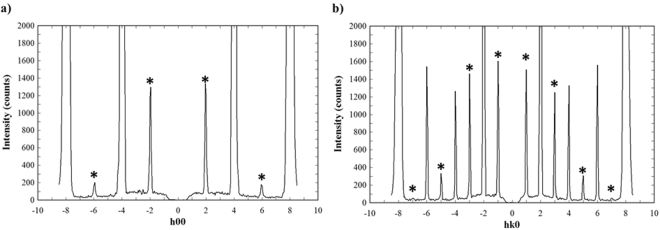
(**a**) h00 line scans in which h is scanned from −8.5 to 8.5 in 0.05 steps. (**b**) hk0 line scans in which h and k are scanned from −8.5 to 8.5 with h = k. Reflections marked with an asterisk indicate reflections that do not obey the extinction rule of *Ia*-3*d* space group.

Although the cubic garnet-type structure is well known as the space group *Ia*-3*d*, it should be noted that the present single-crystal X-ray diffraction intensities detected weak reflections that do not obey the extinction rule of the *Ia*-3*d* space group, as shown in Fig. 4. Concerning indices not following the extinction rule, the space group was confirmed by investigating detailed intensity data with a four circle diffractometer having a scintillation counter. Figure 4(a,b) show h00 line scans in which h is scanned from −8.5 to 8.5 in 0.05 steps and hk 0 line scans in which h and k are scanned from −8.5 to 8.5 with h = k. When the extinction rule of *Ia*-3*d* is h = 4 n at h00 and both h and k with h = k are even at hk0, the intensity is observed. It can be seen from Supplementary Fig. S3 that the intensity is observed at h = 2 n at h 00 and h + k = 2 n at hk 0 (Peak marked with asterisks in Supplementary Fig. S3). Furthermore, these reflections were also observed in single-crystal neutron diffraction. Considering the space group that satisfies all of the observed reflections, space group *I*2_1_3 should be selected for the present single-crystal specimen. This space group was previously described in the garnet-type Li_5_La_3_Ta_2_O_12_^16^. It is very interesting to say that the single-crystal sample of Li_5_La_3_Ta_2_O_12_ was also synthesized at high temperature (1673 K). In the structure refinement that follows, we selected the space group *Ia*-3*d* because of the very low intensity of the additional reflections (1/1000 of the main reflections).

**Figure 5:**
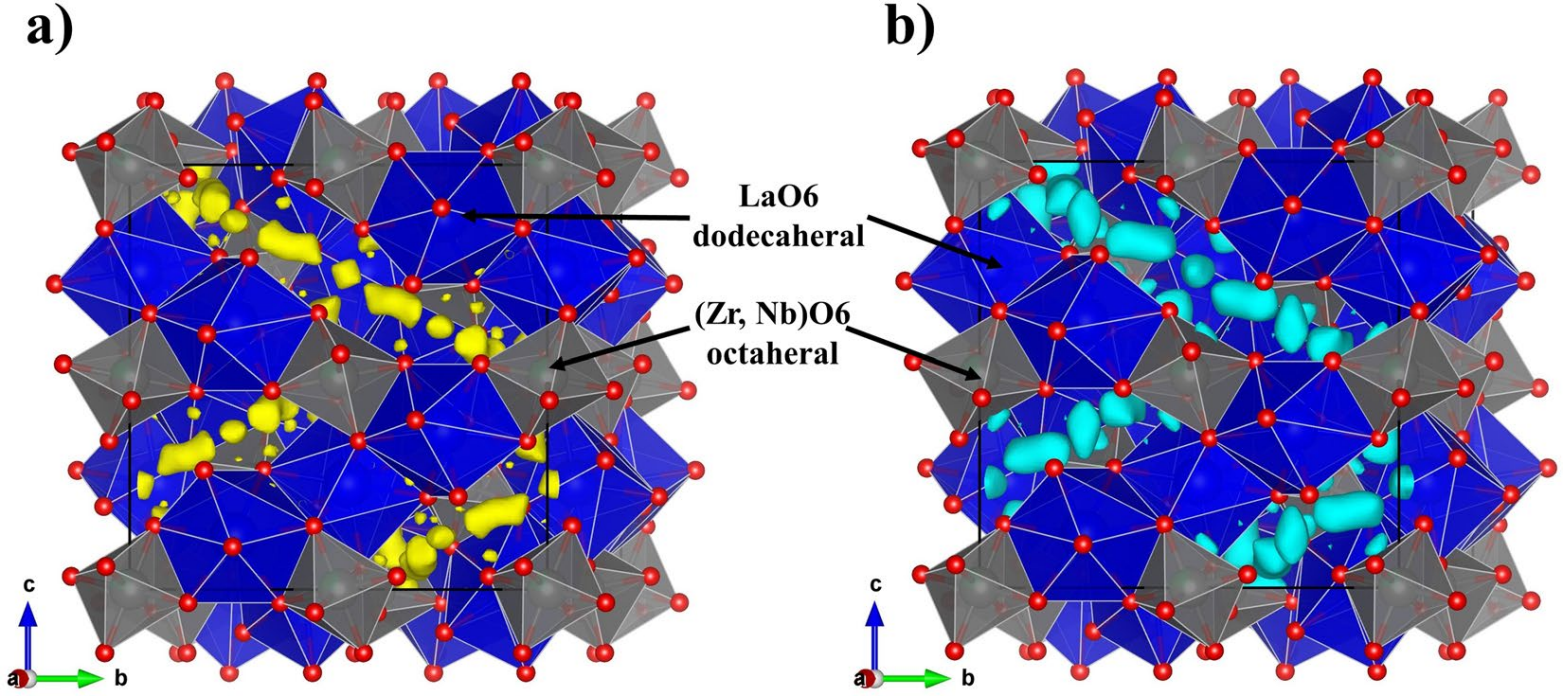
(**a**,**b**) Shows three-dimensional difference Fourier synthesis maps and the (La_3_Zr_1.5_Nb_0.5_O_12_)^6.5−^ framework structure in Li_6.5_La_3_Zr_1.5_Nb_0.5_O_12_. The solid box indicates the unit cell. (**a**) Shows the electron density distributions of the threshold value of 0.45 Å^−3^ in the map prepared from the X-ray diffraction measurement data. (**b**) Shows the nuclear scattering lengths density distributions of the threshold value of 0.3 rm Å^−3^ in the map prepared from the X-ray diffraction measurement data.

**Figure 6:**
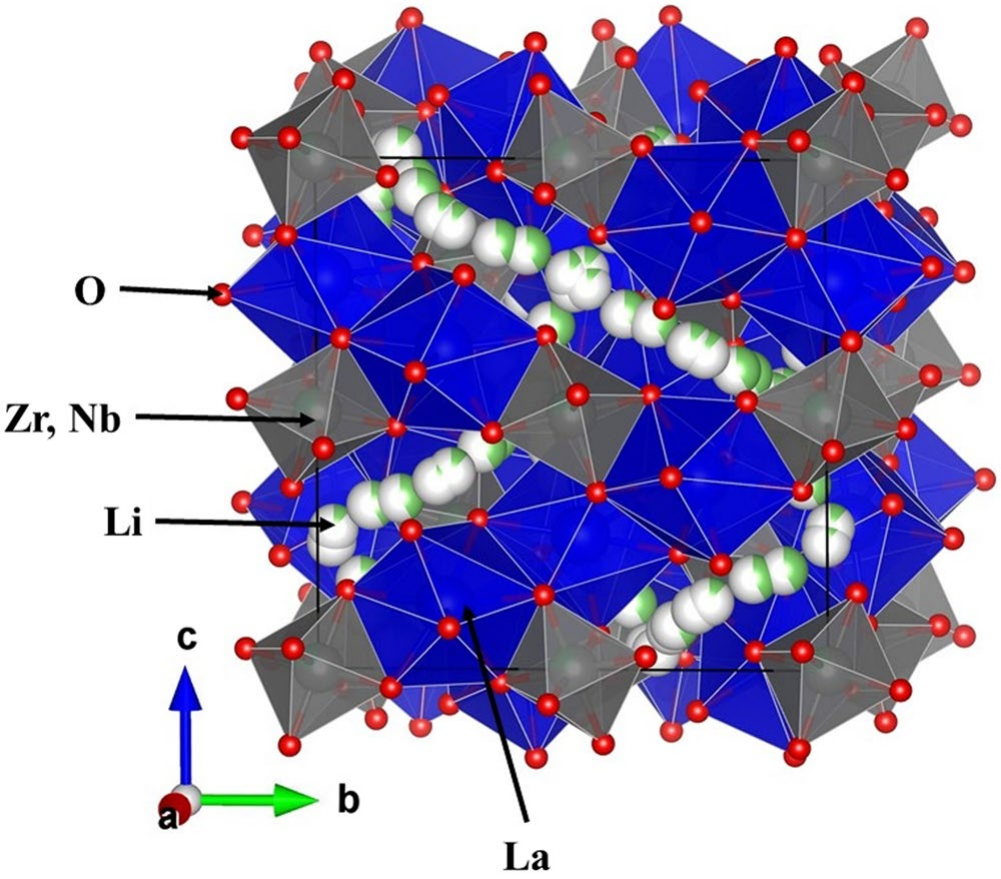
Crystal structure of Li_6.5_La_3_Zr_1.5_Nb_0.5_O_12_ (LLZNb05).

**Figure 7:**
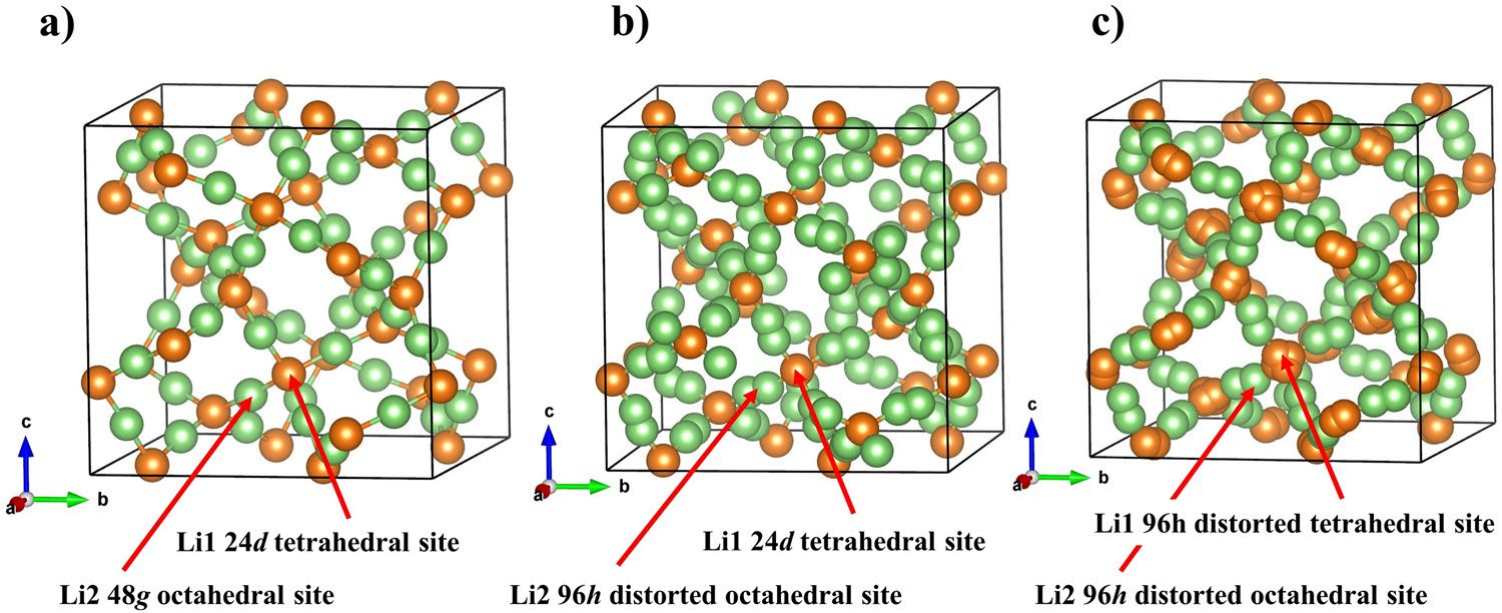
Change in arrangement of lithium ions by each models. (**a**) Crystal structure model in which Li-ions are completely ordered arrangement. (**b**) Crystal structure model in which a part of Li-ions are disordered arrangement. This model is the most reported crystal structure. (**c**) Crystal structure model in which Li-ions are completely disordered arrangement. This model is the most reported crystal structure. This is the crystal structure model shown in this article.

The framework structure consisting of LaO_8_ and (Zr,Nb)O_6_ polyhedra in LLZNb05 was refined using the single-crystal X-ray diffraction data with the reported garnet-type atomic coordinates^14^. The lithium sites were then determined using the difference Fourier synthesis map with both the single-crystal X-ray and neutron diffraction data. The difference Fourier synthesis maps are shown in Fig. 5. Difference Fourier syntheses using the final atomic parameters revealed no significant residual peaks. The resultant reliability values were *R* = 4.25% and w*R* = 5.82% for single-crystal X-ray diffraction data, and *R* = 7.09% and w*R* = 7.94% for single-crystal neutron diffraction data. In this refinement, it is difficult to determine the occupancy ratio of Zr and Nb even if X-rays and neutrons are used complementarily, so the crystal structure was refined using the fixed value determined by ICP-AES. The lattice parameter for the LLZNb05 crystal was refined by the least-squares method using the single X-ray diffraction data (*a* = 12.9130(8) Å). The final atomic coordinates and atomic displacement parameters determined by the single-crystal neutron diffraction data are listed in Table 1. The selected bond distances are shown in Table 2. The distance between Li 1 and Li 2 was 1.42 (9) Å. The refined crystal structure is shown in Fig. 6. The chemical composition was determined as Li_6.45_La_3_Zr_1.5_Nb_0.5_O_12_ by the present structure refinement, which agreed well with the chemical composition obtained from ICP-AES. The Li atoms occupied two interspace sites constructed by the framework structure. The Li1 and Li2 atoms located in the distorted tetrahedral 96 *h* site and distorted octahedral 96 *h* site. In the garnet-type solid electrolyte, the Li site is occupied in the space of the (La_3_Zr_1.5_Nb_0.5_O_12_)^−6.5^ framework structure, which consists the A site formed by (Zr, Nb)O6 and the C site formed by the LaO8. A 96 *h* site forming a distorted tetrahedron occupied by Li ion is a site where 24*d* sites forming tetrahedral sites split into four sites. On the other hand, the 96 *h* site forming a distorted octahedral occupied by Li ion is a site where 48 *g* sites forming an octahedral sites split into two sites. Since the two kinds of Li sites are split together, the distance between the Li ions is shorter than usual, which is considered to contribute to the improvement of Li-ion conductivity. Changes in Li ion arrangement are shown in Fig. 7. This result suggested that the arrangement of lithium ions differed from those in the reported garnet-type structure. In fact, two types of lithium atoms in the crystal structure occupied the tetrahedral 24*d* sites and the distorted octahedral 96 *h* sites in the space group *Ia*-3*d* in the previous report^14,15^. The different degree of the Li site disordering may be affected by the different synthetic temperature. As a result, the Li-Li distances in the present structure were shorter than those in the previous reports. The short Li-Li distance, Li site disordering, and partial occupation of the Li atoms were reported as a key role of lithium ion diffusion in the garnet-type structure.

**Table 1:** Atomic coordinates and equivalent isotropic displacement parameters (Å^2^) for LLZNb05 determined using the single-crystal neutron diffraction data.

Atom	Site	Occ.	*x*	*y*	*z*	*U*_eq_
Li1	96 *h*	0.117 (7)	0.759 (6)	0.112 (5)	−0.019 (8)	0.036 (14)
Li2	96 *h*	0.419 (9)	0.6542 (5)	0.1724 (5)	0.0617 (5)	0.0169 (18)
La1	24*c*	1	0.25	0.125	0	0.00409 (17)
Zr1	16*a*	0.75*	0.25	0.25	0.25	0.00237 (11)**
Nb1	16*a*	0.25*	0.25	0.25	0.25	0.00237 (11)**
O1	96 *h*	1	0.19626 (6)	0.28129 (6)	0.10137 (6)	0.00741 (18)

^*^Fixed to the chemical analysis result by ICP-AES. **Restriction of *U*_eq_(Nb1) = *U*_eq_(Zr1).

**Table 2:** Selected bond distances (Å) for LLZNb05 determined using the single-crystal neutron diffraction data.

La1-O1 × 4	2.5036 (8)	La1-O1 × 4	2.5844 (8)
(Zr1, Nb1)-O1 × 6	2.0805 (8)		
Li1-O1	1.65 (8)	Li1-O1	1.92 (8)
Li1-O1	2.00 (8)	Li1-O1	2.15 (9)
Li2-O1	2.085 (6)	Li2-O1	2.255 (6)
Li2-O1	2.731 (6)	Li2-O1	2.679 (6)
Li2-O1	2.162 (6)	Li2-O1	1.826 (6)
Li1-Li1	0.50 (12)	Li2-Li2	0.809 (8)
Li1-Li2	1.42 (9)

**Figure 8:**
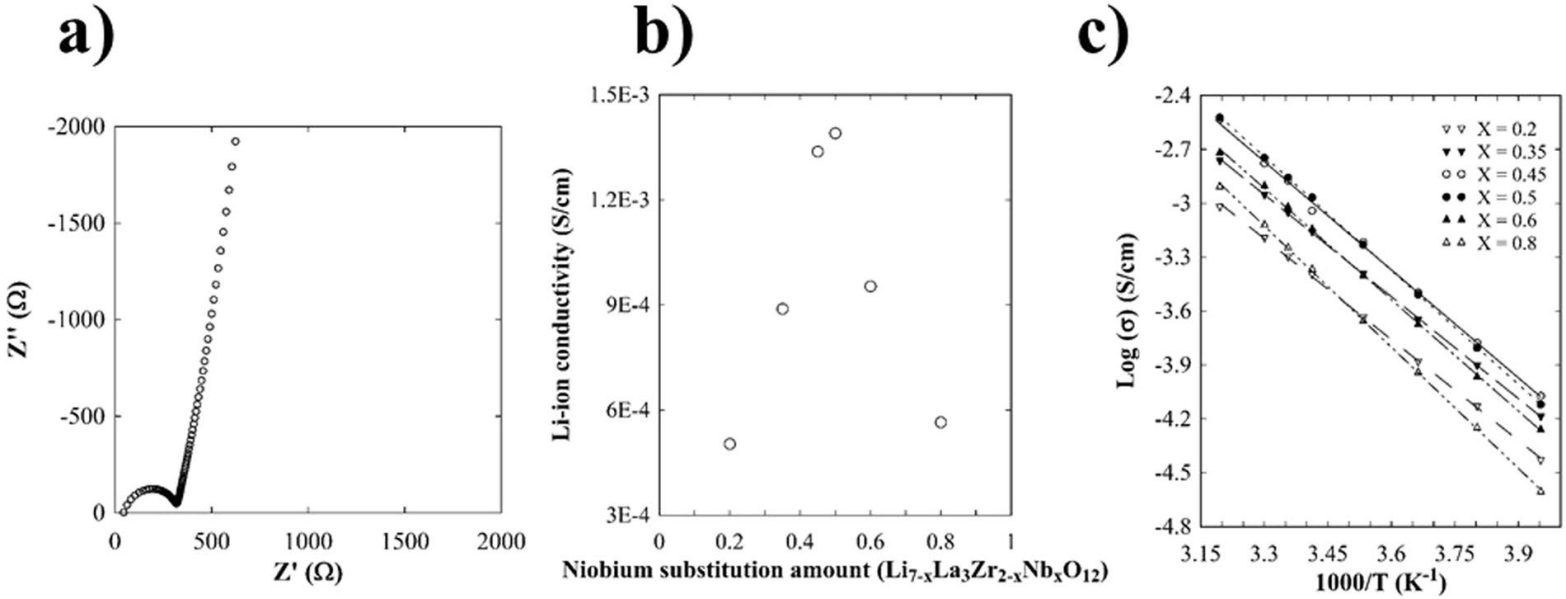
(**a**) AC impedance Nyquist plot of the LLZNb05 single-crystal plate specimen at 298 K in an N_2_ atmosphere. (**b**) Relationship between lthium-ion conductivity at 298 K and the substituted Nb content in the compositional range of Li_7−x_La_3_Zr_2−x_Nb_x_O_12_ with x = 0.2, 0.35, 0.45, 0.5, 0.6, and 0.8. (**c**) Relationship between lithium ion conductivity and temperature of the LLZNb05 single-crystal plate specimen.

Figure 8(a) presents an AC impedance Nyquist plot of the LLZNb05 single-crystal plate samples at 298 K in an N_2_-gas flow atmosphere. The tail of the Nyquist plot at the low-frequency side indicates the blocking of the electrodes for mobile Li ions. The Nyquist plots at the high-frequency side exhibit single semi-circle behavior. Therefore, the conductivity could not be separated into bulk and grain boundaries in the present experiments. The value of Z’ (Ω) at the end of the circle was 330.68 Ω at 298 K. Therefore, the total lithium-ion conductivity in LLZNb05 was 1.39 × 10^−3^ S cm^−1^ at 298 K. This value is comparable to the bulk component measured using the sintered LLZNb05 sample, although the total conductivity of the sintered sample was reported to be 8.0 × 10^−4^ S cm^−1^ at 303 K^8^. This fact indicates that the present single-crystal sample really exhibits only the bulk component of conductivity and does not have grain boundary characteristics such as polycrystalline materials. Figure 8(b) presents the relationship between lithium-ion conductivity at 298 K and the substituted Nb-content in the compositional range of Li_7−x_La_3_Zr_2−x_Nb_x_O_12_ with x = 0.2, 0.35, 0.45, 0.5, 0.6, and 0.8. From this figure, it can be understood that the lithium-ion conductivity was maximum around the Nb content of x = 0.5. Figure 8(c) presents a graph of the relationship between lithium ion conductivity and temperature of the LLZNb05 single-crystal plate samples in the temperature range from 253 K to 313 K. The activation energy was determined to be 0.45 eV from the average rate of change of the Arrhenius plot (Ln (σT) v. s. 1/T). Even in the single-crystal samples with different amounts of niobium substitution, measured activation energy ranged from 0.40 eV to 0.45 eV.

**Figure 9:**
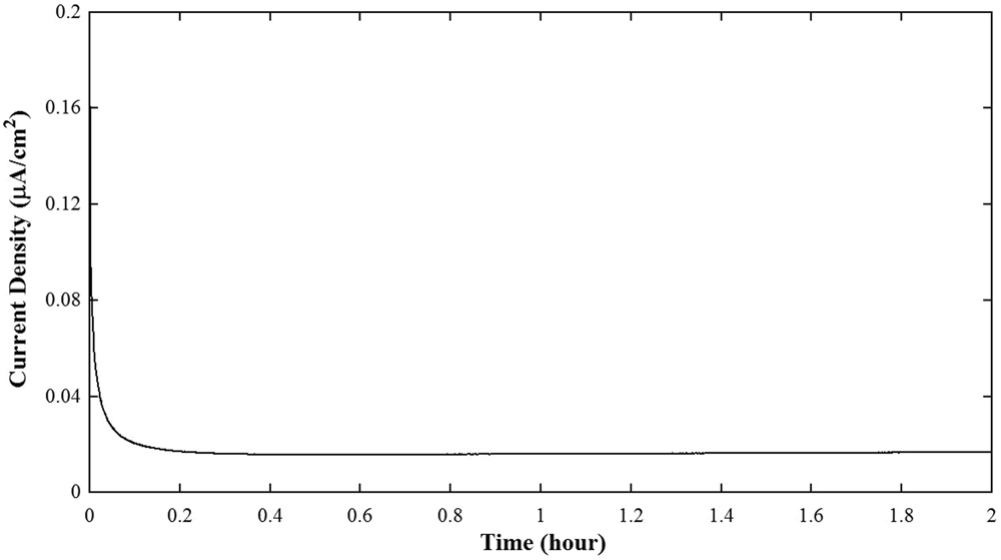
DC polarization measurement of the LLZNb05 single-crystal plate sample at 298 K with two gold blocking electrodes.

**Figure 10:**
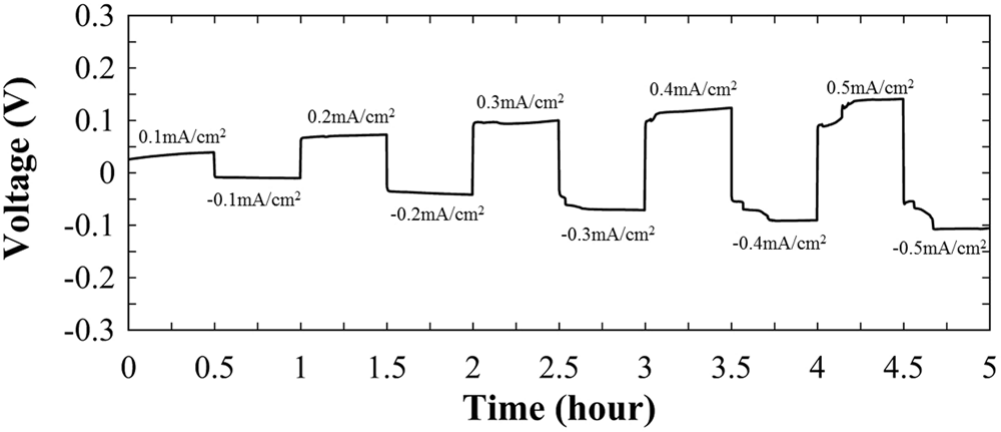
DC polarization measurement on symmetric Li/LLZNb05/Li cells at 298 K.

**Figure 11:**
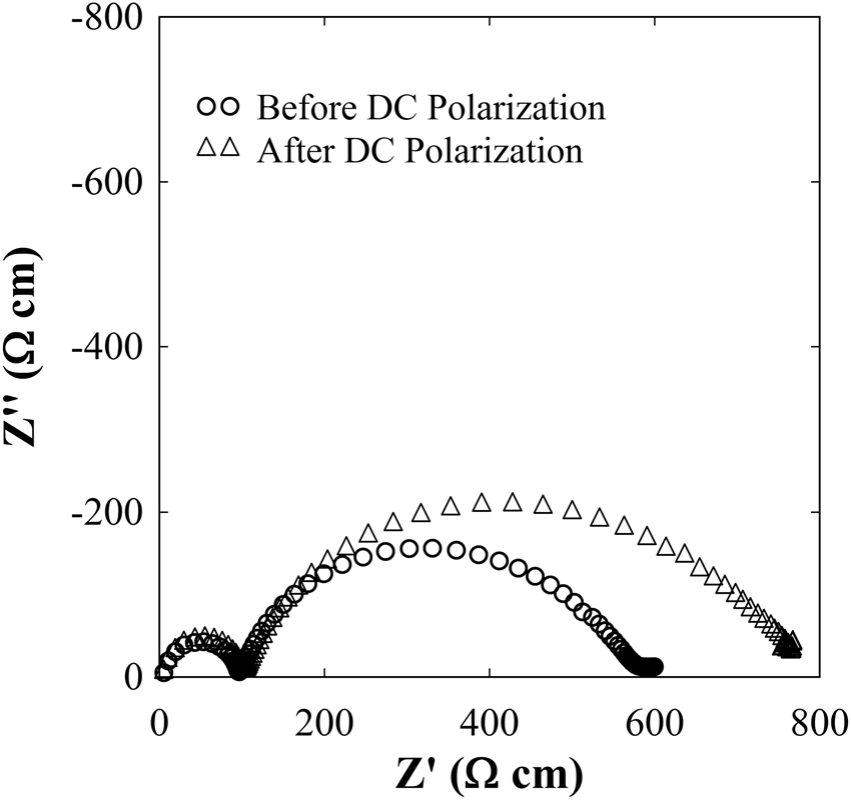
AC impedance measurement of the symmetric Li/LLZNb05/Li cell before and after the DC polarization measurement at 298 K.

Figure 9 illustrates the DC polarization measurement of the LLZNb05 single-crystal plate samples at 298 K with two gold blocking electrodes. When using gold blocking electrodes, the current initially decreases with time due to the polarization, then it becomes almost constant. From the Fig. 9, the result of calculating the electronic conductivity was 7.1 × 10^−6^ S cm^−1^. Therefore, the electron conductivity is very low, and its transport rate is estimated at 0.55%. The short-circuit experiment data on symmetric Li/LLZNb05/Li cells at 298 K obtained by DC polarization measurement is shown in Fig. 10. This result suggests that the LLZNb05 single-crystal plate functions as a solid electrolyte and a separator without short-circuiting to 0.5 mA cm^−2^, and the lithium metal dissolution and deposition reaction repeats reversibly. This means that the dendrite formed did not penetrate the solid electrolyte because the single crystal plate was very dense. As can be seen from Fig. 10, no short-circuit is observed, but voltage behavior is unstable at 0.4 mA cm^−2^ and more. This unstable cause may be the interface contact due to lithium deposition and desorption. Furthermore, the conductivity estimated from DC polarization at 0.1 mA/cm^−2^ was 1.0 × 10^−3^ S cm^−1^. This value is lower than the total conductivity of 1.4 × 10^−3^ S cm^−1^ obtained by AC impedance measurement. Figure 11 shows the results of AC impedance measurement of the symmetric Li/LLZNb05/Li cell before and after the DC polarization measurement at 298 K. The total resistance of the symmetric Li/LLZNb05/Li cell increased by about 600 Ω cm before DC polarization experiment and about 800 Ω cm after the DC polarization experiment. If the symmetrical cell is short-circuited, the resistance will be small, so the arc of the AC impedance should be smaller.

**Figure 12:**
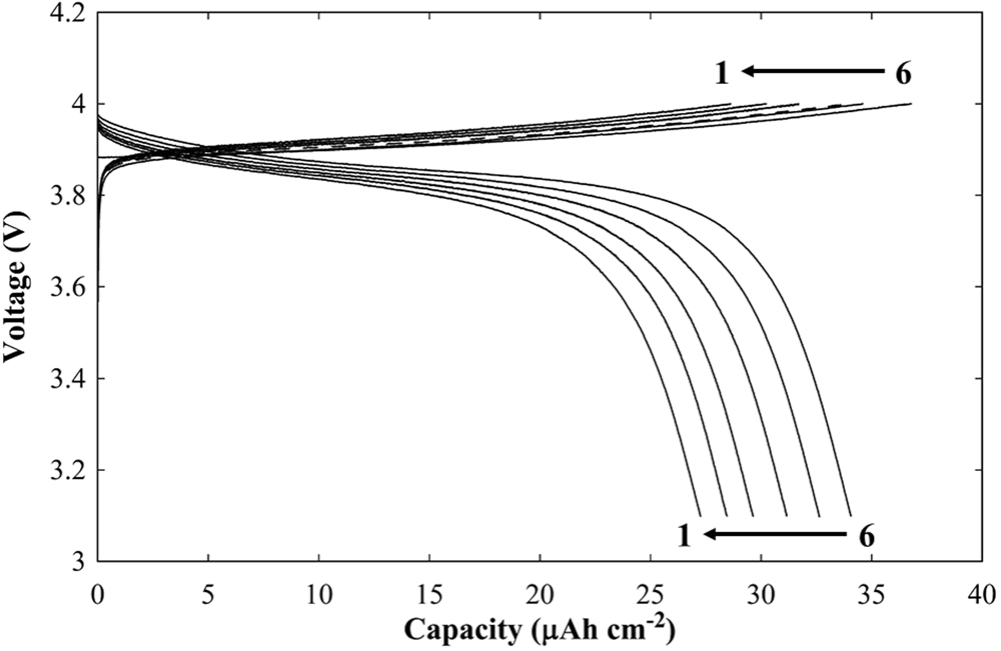
Charge-discharge cycling performance for the Li/LLZNb05/LiCoO_2_ all-solid-state secondary lithium battery at voltages ranging between 3.1 and 4.0 V (vs. Li/Li^+^) at a current density of 8.8 μA cm^−2^ at 353 K.

Figure 12 presents the cycling performance of the charge and discharge tests for the Li/LLZNb05/LiCoO_2_ cell at voltages between 3.1 and 4.0 V at a constant current density of 8.8 μA cm^−2^ at 333 K. Coulomb efficiency of each cycle was 1st 98.4%, 2nd 88.7, 3rd 92.0%, 4th 93.5%, 5th 94.1%, 6th 95.3%, respectively. It is considered that the battery capacity is small because the upper limit of the cutoff voltage is 4.0 V. Although the battery of this time has many problems because it consists only of active material for positive electrode, we think that many problems can be improved by combining solid electrolyte and conductive material.

## Conclusion

We are the first to successfully grow centimeter-sized single crystals of garnet-type Li_6.5_La_3_Zr_1.5_Nb_0.5_O_12_ (LLZNb05) by the FZ method. A detailed crystal structure analysis using single-crystal X-ray and single-crystal neutron diffraction data revealed that Li ions occupied two kinds of 96 *h* sites in the interspaces of the garnet-type framework structure. The resultant shorter Li-Li distance and deficient Li-site arrangement on the conduction pathways constructing the loop structure promised to give the highest lithium-ion conductivity of the present single-crystal specimens. In fact, the AC impedance measurements using single crystal confirmed the lithium ion conductivity of the bulk body as a whole was 1.39 × 10^−3^ S cm^−1^ at 298 K, the highest value among the reported with the same chemical composition. This value relating to the lithium ion migration are higher than those in previous reports using polycrystalline samples. From the results of these chemical and structural characterizations, the garnet-type single-crystal sample has an advantageous bulk nature to realize bulk conductivity without grain boundaries such as in a sintered polycrystalline body. We also performed a short-circuit test with a symmetric cell of Li/LLZNb05/Li using an LLZNb05 single crystal plate as a solid electrolyte, and confirmed that there were no internal short-circuits because the penetration of metallic lithium by dendrites growth was prevented even at 0.5 mA cm^−2^. Finally, we demonstrated charge and discharge cycling performance of the all-solid-state secondary lithium battery using a cell configuration of Li/LLZNb05/LiCoO_2_ in voltages ranging between 3.1 and 4.0 V at 353 K. Accordingly, the garnet single-crystal functions as a solid electrolyte because it is a lithium-ion conductor and electric separator. This all solid state battery still has many problems, but it will be solved in the future.

## Method

### Synthesis

The feedstock polycrystalline materials were first prepared by a conventional solid-state reaction. A mixture of Li_2_CO_3_ (99.9% pure), La_2_O_3_ (99% pure), ZrO_2_ (98% pure), and Nb_2_O_5_ (99% pure) at a molar ratio of 3.9: 1.5: 0.75: 0.25 was heated at 1123 K for 60 h in air. The nominal feedstock composition is Li_7.8_La_3_Zr_1.5_Nb_0.5_O_12_, which is 20% lithium excess composition of the objective Li_6.5_La_3_Zr_1.5_Nb_0.5_O_12_ (LLZNb05), so as to prevent the compositional deviation due to volatilization of lithium during crystal growth. The sample was reground at 200 rpm for 120 minutes by filling a sample, isopropanol and 4 mm zirconia ball in a zirconia pot using a planetary ball mill (P-7, Fritsch GmbH). The feedstock powder filled a rubber tube and formed a cylindrical shape 12 mm in diameter and 83 mm long by isostatic pressing. The rods were subsequently sintered at 1423 K for 4 h in air. The single-crystal growth experiments were performed in air-flow atmosphere using the floating zone (FZ) method in an optical image furnace (Crystal System, Inc.) equipped with four 1000 W halogen lamps.

### Sample characterization

The orientation of as-grown single crystal was measured using a two axis X-ray diffractometer equipped with a CCD detector (Rigaku SmartLab) using monochromatized Cu Kα_1_ radiation (40 kV, 30 mA) by a Johansson’s Ge(111) curved crystal at 295 K. The surface of the single-crystal plate was observed with a scanning electron microscope (JEOL JCM-6000). The chemical compositions of the crystals were analyzed by inductively coupled plasma-atomic emission spectroscopy (ICP-AES, Thermo Jarrell Ash Inc., IRIS Advantage). The pulverized single crystal was dissolved in hydrofluoric acid at 393 K in an autoclave to prepare a solution.

To examine the space group and refine the crystal structure, the single-crystal X-ray diffraction intensity data was measured by a single-crystal X-ray four-circle diffractometer with a scintillation counter (Rigaku AFC-7s) using graphite-monochromatized Mo Kα radiation (50 kV, 30 mA) at 295 K. The single-crystal neutron diffraction was measured with a single-crystal time of flight neutron diffractometer with a 2D detector (J-PARC, BL18, SENJU) using a poisoned decoupled hydrogen moderator (100 kW) at 295 K. X-ray diffraction measurement and neutron diffraction measurement were performed on the same sample processed into a spherical shape with a diameter of 400 micrometers. The structure was refined using computer program Jana2006^21,22^.

### Lithium-ion conductivity

The AC impedance for LLZNb05 single crystals was measured using a Solartron 1260 impedance analyzer operating at 10 mV applied AC amplitude at 13 MHz −10 Hz frequencies at temperature intervals (253 K, 263 K, 273 K, 283 K, 293 K, 298 K, 303 K, and 313 K) in N_2_ gas flow. In the present study, the measurement was carried using a thin-plate specimen having a diameter of 6 mm and a thickness of 1.3 mm obtained by cutting a single-crystal rod. The blocking electrode was 6 mm diameter sputtered gold formed by magnetron sputter deposition.

The DC polarizations for LLZNb05 were measured using lithium foils as non-blocking electrodes and gold formed by magnetron sputter deposition as blocking electrodes at 298 Kusing a Solartron modulab electrochemical analyzer. The 2 mm diameter the blocking electrodes were fabricated on a diameter of 8 mm and thickness of 0.6 mm-thick LLZNb05 single-crystal plate. The 2 mm diameter non-blocking electrodes were fabricated on a diameter of 8 mm and thickness of 3.5 mm-thick LLZNb05 single-crystal plate. In the measurement using Au/LLZNb05/Au, symmetric cells were fabricated by two electrode flat cells, a 200 mv polarization voltage was applied, and the contribution of electron conduction was investigated. In the measurement using Li/LLZNb05/Li, symmetric cell was fabricated by a two-electrode flat cell, the voltage was measured with current densities of 0.1 mA cm^−2^, 0.2 mA cm^−2^, 0.3 mA cm^−2^, 0.4 mA cm^−2^, 0.5 mA cm^−2^ with an applied time of 30 minutes three measurements each current density. The internal short-circuit test of the LLZNb05 single-crystal plate was performed according to this method.

The AC impedance for Li/LLZNb05/Li, symmetric cell before and after the DC polarization measurement were measured using a Solartron 1260 impedance analyzer operating at 100 mV applied AC amplitude at 13 MHz −300 Hz frequencies at 298 K.

### Charge-discharge measurements of all-solid-state lithium battery

Electrochemical charge and discharge tests of the all-solid-state secondary lithium battery were performed using a two electrode flat cell. The solid electrolyte was a single-crystal plate with a diameter of 6 mm and a thickness of 0.7 mm, which were obtained by cutting a single-crystal rod. The positive electrode of LiCoO_2_ was formed on the solid electrolyte by the sol-gel method as follows. First, 0.02 mol kg^−1^ precursor solution was prepared by dissolving lithium acetate and cobalt acetate at a molar ratio of 1:1 using ethylene glycol as a solvent. Next, 10 μl of the precursor solution was spin-coated onto the solid electrolyte five times and then it was heated at 1123 K for 10 minutes in air. The negative electrode was a 5 mm diameter lithium foil. Cells were constructed in an argon-filled glove box, and electrochemical measurements were carried out at a constant current density of 8.8 μA cm^−2^ at voltages between 3.1 and 4.0 V at 353 K after standing for 24 hour in an open circuit condition.

## Electronic supplementary material

Result of checkcif

Result of checkcif
