# Supplementary material for: Lithium-ion conducting oxide single crystal as solid electrolyte for advanced lithium battery application
Source: Sci Rep. 2018 Jul 2;8:9965. doi: 10.1038/s41598-018-27851-x (PMC6028625; doi:10.1038/s41598-018-27851-x)

checkCIF/PLATON report

You have not supplied any structure factors. As a result the full set of tests cannot be run.

THIS REPORT IS FOR GUIDANCE ONLY. IF USED AS PART OF A REVIEW PROCEDURE FOR PUBLICATION, IT SHOULD NOT REPLACE THE EXPERTISE OF AN EXPERIENCED CRYSTALLOGRAPHIC REFEREE.

No syntax errors found. CIF dictionary Interpreting this report

Datablock: Li6.5La3Zr1.5Nb0.5O12

Bond precision: La- O = 0.0030 A Wavelength=0.71069

Cell:	a=12.9130(8)	b=12.9130(8)	c=12.9130(8)
	alpha=90	beta=90	gamma=90
Temperature:	298 K		

	Calculated	Reported
Volume	2153.2(4)	2153.2(2)
Space group	I a -3 d	I a -3 d
Hall group	-I 4bd 2c 3	-I 4bd;2ab;
Moiety formula	La4 Li1.87 Nb0.67 O16 Zr2, 3.353(Li2)	?
Sum formula	La4 Li8.57 Nb0.67 O16 Zr2	La3 Li6.429 Nb0.5 O12 Zr1.5
Mr	1115.52	836.60
Dx,g cm-3	5.162	5.162
Z	6	8
Mu (mm-1)	13.626	13.626
F000	2934.3	2934.0
F000’	2884.33	
h,k,lmax	35,35,35	17,23,35
Nref	1922	1739
Tmin,Tmax		0.034,0.128
Tmin’		

Correction method= # Reported T Limits: Tmin=0.034 Tmax=0.128
AbsCorr = NUMERICAL

Data completeness= 0.905 Theta(max)= 75.880

R(reflections)= 0.0424(1038) wR2(reflections)= wR= 0.0667(1739)

S = 1.570 Npar= 24

The following ALERTS were generated. Each ALERT has the format
test-name_ALERT_alert-type_alert-level.
Click on the hyperlinks for more details of the test.

Alert level C

```
ABSTY02_ALERT_1_C  An _exptl_absorpt_correction_type has been given without
            a literature citation. This should be contained in the
            _exptl_absorpt_process_details field.
            Absorption correction given as numerical
PLAT041_ALERT_1_C Calc. and Reported SumFormula    Strings  Differ     Please Check
PLAT127_ALERT_1_C Implicit Hall Symbol  Inconsistent with Explicit    -I 4bd;2ab;3
```

Alert level G

```
PLAT005_ALERT_5_G No Embedded Refinement Details found  in the CIF     Please Do !
PLAT045_ALERT_1_G Calculated and Reported Z Differ by a Factor ...       0.75 Check
PLAT068_ALERT_1_G Reported F000 Differs from Calcd (or Missing)...     Please Check
PLAT152_ALERT_1_G The Supplied and Calc. Volume s.u. Differ by ...          2 Units
PLAT180_ALERT_4_G Check Cell Rounding: # of Values Ending with 0 =          3 Note
PLAT232_ALERT_2_G Hirshfeld Test Diff (M-X)  La1    --  O1      ..        6.3 s.u.
PLAT300_ALERT_4_G Atom Site Occupancy of >Zr1    is Constrained at       0.75 Check
PLAT300_ALERT_4_G Atom Site Occupancy of <Nb1    is Constrained at       0.25 Check
PLAT300_ALERT_4_G Atom Site Occupancy of <Li1    is Constrained at     0.1167 Check
PLAT300_ALERT_4_G Atom Site Occupancy of <Li2    is Constrained at     0.4191 Check
PLAT301_ALERT_3_G Main Residue  Disorder ..............(Resd  1)..         23 % Note
PLAT302_ALERT_4_G Anion/Solvent/Minor-Residue Disorder (Resd  2)..        100 % Note
PLAT304_ALERT_4_G Non-Integer Number of Atoms (  24.53) in Resd. #          1 Check
PLAT304_ALERT_4_G Non-Integer Number of Atoms (   0.84) in Resd. #          2 Check
PLAT808_ALERT_5_G No Parseable SHELXL Style Weighting Scheme Found     Please Check
PLAT811_ALERT_5_G No ADDSYM Analysis: Too Many Excluded Atoms ....          ! Info
PLAT881_ALERT_1_G Missing datum for _diffrn_reflns_av_R_equivalents    Please Check
PLAT950_ALERT_5_G Calculated (ThMax) and CIF-Reported Hmax Differ          18 Units
PLAT951_ALERT_5_G Calculated (ThMax) and CIF-Reported Kmax Differ          12 Units
```

0 **ALERT level A** = Most likely a serious problem - resolve or explain
0 **ALERT level B** = A potentially serious problem, consider carefully
3 **ALERT level C** = Check. Ensure it is not caused by an omission or oversight
19 **ALERT level G** = General information/check it is not something unexpected

7 ALERT type 1 CIF construction/syntax error, inconsistent or missing data
1 ALERT type 2 Indicator that the structure model may be wrong or deficient
1 ALERT type 3 Indicator that the structure quality may be low
8 ALERT type 4 Improvement, methodology, query or suggestion
5 ALERT type 5 Informative message, check

It is advisable to attempt to resolve as many as possible of the alerts in all categories. Often the minor alerts point to easily fixed oversights, errors and omissions in your CIF or refinement strategy, so attention to these fine details can be worthwhile. In order to resolve some of the more serious problems it may be necessary to carry out additional measurements or structure refinements. However, the purpose of your study may justify the reported deviations and the more serious of these should normally be commented upon in the discussion or experimental section of a paper or in the "special_details" fields of the CIF. checkCIF was carefully designed to identify outliers and unusual parameters, but every test has its limitations and alerts that are not important in a particular case may appear. Conversely, the absence of alerts does not guarantee there are no aspects of the results needing attention. It is up to the individual to critically assess their own results and, if necessary, seek expert advice.

Publication of your CIF in IUCr journals

A basic structural check has been run on your CIF. These basic checks will be run on all CIFs submitted for publication in IUCr journals (*Acta Crystallographica*, *Journal of Applied Crystallography*, *Journal of Synchrotron Radiation*); however, if you intend to submit to *Acta Crystallographica Section C* or *E* or *IUCrData*, you should make sure that full publication checks are run on the final version of your CIF prior to submission.

Publication of your CIF in other journals

Please refer to the *Notes for Authors* of the relevant journal for any special instructions relating to CIF submission.

PLATON version of 24/11/2016; check.def file version of 23/11/2016

Datablock Li6.5La3Zr1.5Nb0.5O12 - ellipsoid plot

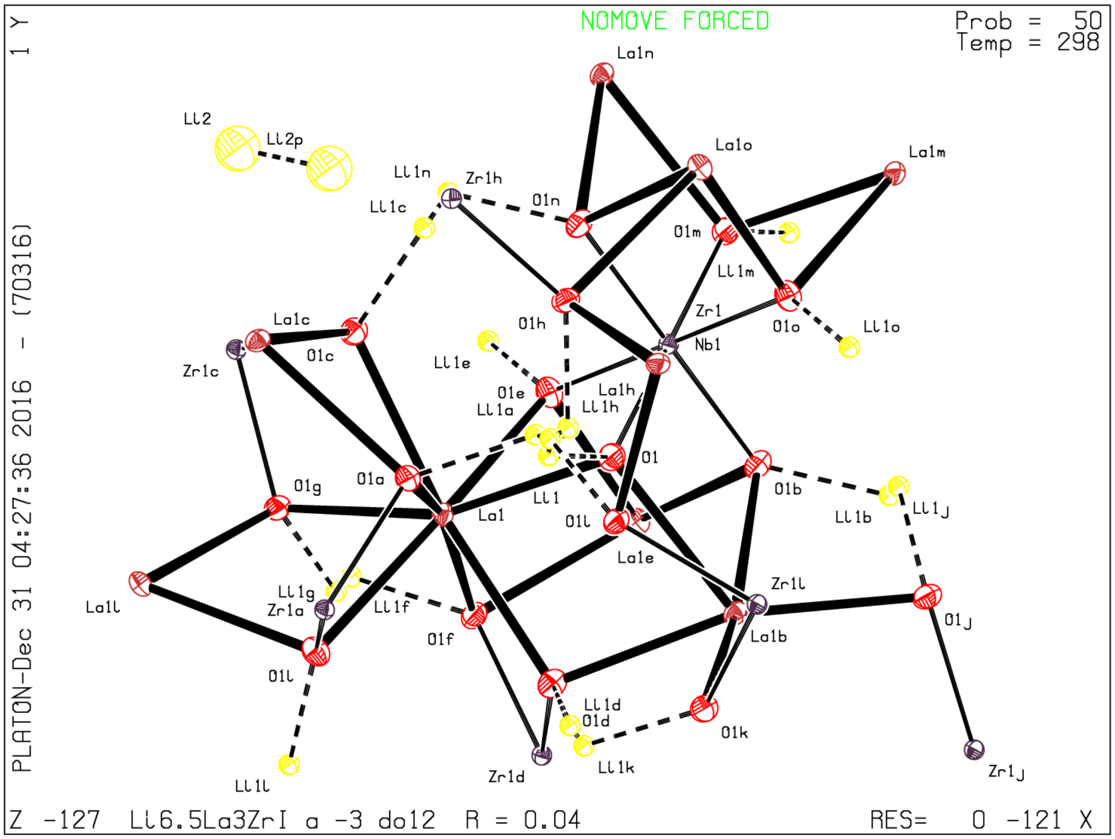

Supplement: Supplementary file 2 — Result of checkcif [file 41598_2018_27851_MOESM2_ESM.pdf]
